# Assessing the price levels of medical service and influential factors: evidence from China

**DOI:** 10.1186/s12889-024-17639-2

**Published:** 2024-01-08

**Authors:** Yanxian Lin, Luo Li, Bao Liu

**Affiliations:** 1 Department of Health Economics, School of Public Health, Fudan University, 130 Dong An Rd, Shanghai, 200032 China; 2 Key Laboratory of Health Technology Assessment (Fudan University), Ministry of Health, Shanghai, China

**Keywords:** Medical service, Medical service prices, Price level, Influential factors, China

## Abstract

**Background:**

Medical service prices play a crucial role in cost containment in China. This study aimed to assess the change in medical service price levels at the macro level and the relationship with relevant macroeconomic factors.

**Methods:**

Data from the 2022 China Statistics Yearbook, the 2022 China Health Statistics Yearbook, and the 2020 China National Health Accounts Report were used. Time trends of health price levels, utilization, and health expenditure were examined. A time-series regression model was employed to measure the impact of service utilization and medical service prices on total medical service expenditure growth from 2000 to 2021. The Johansen cointegration test was conducted to test the cointegrating relationship between medical service price levels and total medical service expenditure, average wage of employees and CPI. The Granger causality test was performed to observe the direction of causality.

**Results:**

Descriptive analyses showed consistent growth in utilization and medical service price levels from 2000 to 2021. The time-series model indicated that medical service expenditure was influenced by the rise in inpatient admissions and price levels of medical service and medicine. The Johansen cointegration test identified a long-term equilibrium relationship between medical service price levels and total medical service expenditure, average wage and CPI. The change in medical service price levels was the Granger cause of the change in medical service expenditure, but it had no impact on average wage and CPI. However, the change in medical service price levels was influenced by these three macroeconomic factors.

**Conclusions:**

The growth of medical service expenditure in China was driven by inpatient use and price level. There was a long-term equilibrium relationship between medical service price levels and relevant macroeconomic factors. However, medical service price levels only affected medical service expenditure and have no impact on average wage and CPI. It is necessary to improve the value transmission mechanism of medical service prices.

## Background

With the considerable increase in health expenditure, cost containment is one of the most pressing challenges faced by healthcare systems [1]. The health expenditure is driven by volumes, price or their interplay [2]. While some studies suggested that utilization is the primary factor accounting for health expenditure [3, 4], others contended that prices are the main contributing factor [5, 6]. Developed and developing countries have implemented cost containment policies that target price as common strategies to control the rapid growth of health expenditures [7–9]. However, the existing literature predominantly focuses on developed countries, with few studies conducted in developing countries. As a rapidly developing country, China faces substantial challenges in controlling its health expenditure inflation [10]. The healthcare market in China is relatively monopolistic, with public hospitals providing most healthcare service [11]. Since the beginning of this century, China has implemented price regulation policies for public hospitals, which set ceiling prices based on fee schedules to control health expenditure [12]:

In the first phase (2000–2008), the Chinese medical pricing system evolved from government-determined prices to government-guided prices and market-regulated prices. The pricing of medical service was managed through a system in which the central government was responsible for enacting medical service items to regulate the price of items, while provincial authorities determined celling prices for public hospitals. The National Development and Reform Commission was the governing department responsible for formulating the National Medical Service Price Items Standard, while the Provincial Development and Reform Commission set its own guiding prices for medical service.

From 2000 to 2008, Chinese prices management policy mainly focused on revising the standardization of medical service price items, including 3966 items in 2001 and 4170 items in 2007. Since the National Medical Service Price Items Standard in 2001 was the first nationwide unified standard for medical service, provinces concentrated on aligning their provincial items standards with national standards and establishing cost-based prices. Despite proposed cost-based pricing, almost none of the provinces have the capacity to revise medical service prices based on costs. As hospitals were responsible for their own finances and medical service prices were subject to strict prices regulations, hospitals mainly obtained cross revenue from roughly 15% markup on drug prices. However, this practice distorted China’s medical prices management mechanism, and hospitals’ over-prescription and over-treatment behaviors have rapidly increased healthcare expenses [13, 14].

In the second phase (2009–2017), the Chinese prices management policy focused on adjusting medical service prices based on cost and hospital revenue structure. In 2009, China launched a deepening health reform to adjust the revenue of public hospitals, key part of the reform was ending the previous 15% drug markups and the associated increase in medical service prices to promote a more rational medical prices management system in China. The standardization of medical service price items serves as the cornerstone for pricing, and China underwent a comprehensive revision of its medical service pricing items standard in 2012. This revision overcame the shortcomings found in the 2001 and 2007 versions. The classification framework of this revision drew on the standards of the International Classification of Procedure in Medicine (ICPM) established by the World Health Organization and classification systems from countries including the United States, Germany, Canada, and Australia. Moreover, it incorporated labor cost considerations into the item’s scope. The 2012 edition of medical service items encompassed 9,360 indivisible items. In 2012, China initiated the reform of public hospitals. Local governments increased the prices of medical service that reflect the value of the medical staff’s technical service, such as surgeries and nursing, to compensate for the loss of the hospital caused by the cancellation of drug markups. In addition, prices for high-tech diagnostic tests were lowered [9]. During this period, although the prices of medical service were adjusted, the effectiveness of medical service prices reform was limited because medical service prices involved the interests of patients, hospitals, and healthcare security [15, 16], and accurately accounting for the costs of medical service posed challenges [17].

In the third phase (2018 to now), China underwent a national institutional reform and established the National Healthcare Security Administration (NHSA) in 2018, assuming the responsibility for medical prices management. Since then, the focus has shifted towards exploring more effective pricing management mechanisms. Between 2000 and 2022, the share of total health expenditure in Gross Domestic Product (GDP) increased from 4.57 to 7.01% [18], representing a growth of approximately 1.53 times. This phenomenon prompted policymakers to shift their perspective from micro-pricing in the specialized healthcare field to macro-management within the economic and social domains. Previous empirical evidence also demonstrated that solely raising prices failed to meet the expectations of prices management. The healthcare sector is a crucial component of the macroeconomy and involves the interests of various stakeholders. China’s government combined the adjustment of medical service prices with external macroeconomic development by incorporating macroeconomic factors into the pricing adjustment process, such as medical service expenditure, medical insurance fund surplus, GDP, the Consumer Price Index (CPI), and other macroeconomic indicators. In 2021, the National Healthcare Security Administration divided medical service items into general and complex items and adjusted their prices based on the characteristics of each item type and in conjunction with different historical macroeconomic indicators. The administration selected five cities below the first-tier level (Tangshan, Suzhou, Xiamen, Ganzhou and Leshan) to pilot the program for five years.

According to China’s medical service prices reform in the past 20 years mentioned above, the policy of adjusting medical service prices is the focus of medical service prices reform. Therefore, the price levels of medical service has changed due to policies. However, existing research focused on short-term medical service prices reform policies, especially public hospital reform of the cancellation of drug markups and associated adjustment of medical service prices, which had an impact on the changes in medical service prices, medical expenditure, service quantity, quality, and efficiency of public hospitals [19–21]. In addition, previous studies mainly focused on exploring the role of medical service prices from a micro-perspective, and macro-perspective studies are few. The relationship between total health expenditure and macroeconomics has been extensively and deeply studied. Price and quantity are crucial factors driving the increase in health expenditure, it is worth studying the medical service prices from macroeconomics. The Shi et al. discussed the impact of changes in medical service price levels on total drug expenditure from 1990 to 2009 [22]. The Yan et al. discussed the driving effect of changes in medical service prices on medical expenditures from 2008 to 2018 [3]. The Li et al. compared the differences in price levels between different regions and their relationship with macroeconomic development [23]. Medical service expenses serve as the main source of income for medical institutions and healthcare workers, and income is used for consumption. From a macro perspective, changes in medical service prices may have an impact on the relationship between average wage and consumption level. This value transmission relationship is also reflected in the pilot policy for medical service prices reform, which selects average wage of urban employees and CPI as the indicators for adjusting medical service prices. No study, however, has estimated the relationship between medical price levels and related macroeconomic factors in China.

Therefore, in order to better understand the impact of China’s medical service prices reform policies over the past 20 years on the medical service price levels and their macroeconomic transmission effects, this study aims to examine China’s long-term changes in medical service price levels and their influential factors from a macro perspective.

## Methods

### Data source and study variables

This study utilized national aggregate data (2000–2021) sourced from the China Statistics Yearbook, the China Health Statistics Yearbook, and the China National Health Accounts Reports of 2020 [24–26]. The China Statistics Yearbook provides annual data on macroeconomic development throughout the nation and its provinces. The China Health Statistics Yearbook contains annual data on national health statistics, health expenditure, and health development at both the national and provincial levels. The China National Health Accounts Report of 2020 comprises national expenditure data concerning China’s medical service and pharmaceutical expenditure. The method of national health accounts meticulously traced all expenses by applying the OECD system of accounts in conjunction with China’s existing healthcare service delivery system.

In the section examining the medical service price level. Following previous study [22], we assumed that the contributing factors of the annual health expenditures were explained by two factors: utilization and price indices. Our study used the data from the China National Health Accounts Reports to reflect the health expenditures using the accounting method of health providers, including total health expenditures (THE), total hospital expenditures, and total pharmaceutical expenditures (TPE). As data on medical service expenditures were unavailable, this study subtracted the total pharmaceutical expenditures (including outpatient and inpatient) from the total hospital expenditures to serve as a proxy indicator for total medical service expenditures (TMSE). The evaluation also incorporates pharmaceuticals to comprehensively assess medical service price level. In addition, THE, TPE, and TMSE of 2021 were interpolated using the data from 2020. We used the number of inpatient admissions and outpatient visits as proxy variables for utilization and the healthcare price index to represent the price level, including the health care price index (HPI), the medicine price index (MPI), and the medical service price index (MSPI). In the section examining the relationship between medical service price levels and macroeconomic factors, we mainly considered the relationship between MSPI and CPI, average wage of urban employees, and TMSE. To eliminate the effect of inflation, GDP, average wage of urban employees, and all expenditures were converted to the price level of the year 2000 using the GDP deflator in China. To compare the long-term trends changes in price index, all price indices were calculated with 2000 as the base year.

### Statistical analysis

Descriptive methods were used to examine the time trends of THE, TPE, and TMSE. The annual utilization and price levels were examined and plotted to describe the time trends. The determinants of TMSE were examined using two indicators: the volume of healthcare utilization and the price index. We employed a static time series linear regression model to investigate the determinants for TMSE in China. A static model was used for the analyses of annual TMSE data because the length of the observation interval was long enough to address seasonal fluctuation:

1 $$ {TMSE}_{t}={\beta }_{0}+{\beta }_{1}{MSPI}_{t}+{\beta }_{2}{MPI}_{t}+{\beta }_{3}{INPT}_{t}+{\beta }_{4}{OUTPT}_{t}+{u}_{t}$$

where *TMSE* denoted total medical service expenditure in year *t, MSPI*_*t*_ was medical service price index, *MPI*_*t*_ was medicine price index, *INPT*_*t*_ was the number of inpatient admissions, and *OUTPT*_*t*_ was the number of outpatient visits, *u*_*t*_ was the residuals and followed the linear regression model assumptions. In addition, the Durbin–Watson statistic was used to test serial correlation.

The Vector Autoregression (VAR) model was used to investigate the relationship between levels of medical service price and macroeconomic factors. As medical service prices influence exhibits certain time lags and the relationship between medical service price and macroeconomic factors was intricate to disentangle. VAR was considered one of the most flexible models for analyses of multivariate time series that multivariate variables are both explained and explanatory variables [27, 28]. Each variable only depended on the lagged values of all selected variables. The general form of VAR can be written as:

2 $$ {Y}_{t} ={\mu }+{\varPi }_{1}{Y}_{t-1}+\cdots +{\varPi }_{k}{Y}_{t-k}+{\varepsilon }_{t}$$

where ${Y}_{t}$is a (*n* × 1) vector of endogenous variables, $\mu$ represented a *k* × 1 vector of constants (intercept), ${\varPi }_{k}$is the (*n* × *n*) matrix of autoregressive coefficients for *k* = 1, 2,…., p, and ε_t_ = (ε_*1t*_,……., ε_*nt*_)′ is the (*n* × 1) generalization of a white noise process. By employing the VAR-based framework, the Johansen Cointegration test was utilized to examine whether a cointegration relationship exists between the variables, which implies a long-run stable equilibrium relationship between medical service price levels and macroeconomic factors. To further elucidate the underlying causal mechanisms, the Granger causality test was used to determine whether lag variables could predict the current value of another variable. If the effect is significant, there is a Granger causality between this variable and another variable; otherwise, there is no Granger causality.

## Results

### Time trends of medical service price levels

**Fig. 1:**
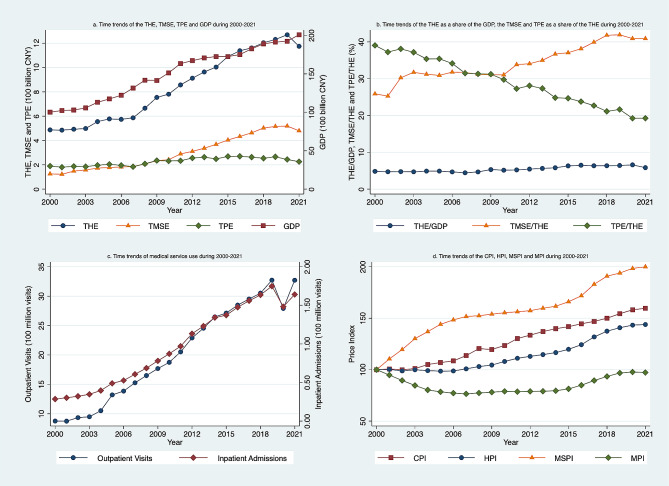
Time trends of the health expenditure and decomposing factors between 2000 and 2021. **a** Left y-axis represents THE (total health expenditure), TMSE (total medical service expenditure), and TPE (total pharmaceuticals expenditure). Right y-axis represents GDP (gross domestic product). The units for both y-axes are in 100 billion Chinese RMB. **b** y-axis refers to percentages. **c** Left y-axis represents the number of outpatient visits. Right y-axis represents the number of hospital admissions. The units for both y-axes are in 100 million visits. **d** y-axis refers to the price index, including CPI (consumer price index), HPI (health care price index), MPI (medicine price index), and MSPI (medical service price index)

Figure 1 presents the annual time trends of health expenditures and related factors during 2009–2021. Figure 1a shows the time trends of GDP, THE, TPE, and TMSE. THE and TMSE displayed growth trends along with GDP, but the growth spread of TPE was slow. Figure 1b presents THE as a share of GDP, TPE and TMSE as a share of THE. From 2000 to 2021, the ratio of THE to GDP fluctuated around 5%, with an increase of 20.30%. The composition of THE between medical service and pharmaceutical expenditures has shifted greatly over time. The ratios of TPE were significantly higher than TMSE before 2007 but lower than TMSE after 2009. Figure 1c presents the time trends of health service use. Both outpatient visits and inpatient visits displayed positive growth trends over the study period. Figure 1d presents the time trends of CPI and health price indices calculated based on the year 2000 from 2009 to 2021. Using 100 as the baseline for measuring changes. CPI exceeding 100 indicates an upward price trend in China over these 21 years. Since 2007, HPI has consistently maintained levels above 100, indicating a continuous rise in the overall price levels of the healthcare industry. According to the price level in 2000, MPI has shown a declining trend, while MSPI has significantly increased, surpassing the overall price inflation levels in the healthcare sector over 21 years.

The results of the determinants of TMSE are presented in Table 1. The time series mode was overall significant (*p* < 0.001) at 0.05 significant level. The R^2^ was 0.991, indicating that 99.1% variation of the growth of TMSE after excluding price factors was explained by the model. TMSE was significantly correlated with price indices (medical service and medicine price index) and health care utilization (inpatient admissions and outpatient visits). The price indices had a significant influence on TMSE, including medical service(*p* = 0.001) and medicine (*p* < 0.001). Inpatient service utilization had a positive effect (0.53, *p* < 0.001), whereas outpatient service use had a negative one (−0.02, *p* = 0.002). The Durbin-Watson test statistic of serial correlation was 1.910, falling into the zone of acceptance of the null hypothesis and suggesting no serial correlation.

**Table 1:** Determinants of TMSE in China

TMSE	Coefficient	SE	P-value
MSPI_t_	18.23	4.28	0.001
MPI_t_	16.07	3.07	<0.001
OUTPT_t_	−0.02	0.01	0.002
INTPT_t_	0.53	0.09	<0.001
_cons	−1868.65	580.19	0.005

INPT_t_ denotes number of inpatient admissions, MPI_t_ denotes medicine price index, MSPI_t_ denotes medical service price index, OUTPT_t_ denotes number of outpatient visits, TMSE denotes total medical service expenditure. SE denotes standard error

### Influential factors of medical service price levels

To mitigate the impact of heteroscedasticity on the model results, a logarithmic transformation was applied to all variables. The prerequisite for establishing the VAR model is that the variables or the differenced sequences of variables meet the requirement of stationarity. We conducted a time series stationarity test on the variables after logarithmic transformation using the Augmented Dickey-Fuller (ADF) and Phillips-Perron (PP) tests. Table 2 summarizes the results of these formal tests, indicating that the second difference of all variables is stationary.

**Table 2:** Unit root test

	Levels		First differences		Second difference	
	ADF test	PP test	ADF test	PP test	ADF test	PP test
ln(MSPI)	−4.316**	−3.038*	−2.522	−2.534	−4.692**	−4.671**
ln(CPI)	0.045	0.087	−3.985**	−3.985**	−5.604**	−7.077**
ln(AW)	−1.526	−1.592	−4.620**	−4.944**	−4.994**	−5.512**
ln(TMSE)	−1.199	−1.195	−4.076**	−4.244**	−9.979**	−10.383**

AW denotes average wage of urban employees, CPI denotes consumer price index, MSPI_t_ denotes medical service price index, TMSE denotes total medical service expenditure

** and * denote the significance at the 1% and 5% level respectively

We employed the Johansen cointegration tests to ascertain a long-term equilibrium relationship among the variables within the model. Table 3 shows that Trace Statistics and Max-Eigen Statistics fail to reject the null hypothesis of cointegration rank = 2 at a 5% significance level. This suggests that there are at most two cointegration relationships among all variables. These results offer more robust evidence of the long-term equilibrium and stable relationship between the medical service price index and its influential factors.

**Table 3:** The Johansen cointegrating test

No. of CE(s)	Trace statistic	5% Critical value	Max-eigen statistic	5% Critical value
*r* = 0	68.398	54.64	30.425	30.33
*r* = 1	37.974	34.55	25.004	23.78
*r* = 2	12.970*	18.17	8.940*	16.87
*r* = 3	4.030	3.74	4.030	3.74

Rejection of the null hypothesis at the 5% level

Given the proven cointegration, the presence of a causal link is expected, so further Granger causality Wald test between the price levels of medical service and three macroeconomic variables in the VAR model was tested. According to the SC minimum principle, the lag period was finally selected as the third order. Table 4 shows that the medical service price levels is the Granger cause of TMSE but not the Granger cause of average wage and CPI. In other words, changes in the medical service price index only have a significant on changes in TMSE, but the impact of changes in average wage and CPI is not obvious. The results of the Granger cause also show that CPI, average wage, and TMSE are the Granger causes of MSPI. This implies that the past movements in macroeconomic factors explain changes in MSPI.

**Table 4:** Granger causality

Null hypothesis	Chi^2^	p-value	Result
MSPI does not Granger Cause TMSE	22.183	<0.05	Reject
MSPI does not Granger Cause AW	2.556	0.465	Accpet
MSPI dose not Granger Cause CPI	0.914	0.822	Accpet
TMSE dose not Granger Cause MSPI	11.167	0.011	Reject
AW does not Granger Cause MSPI	8.7385	0.033	Reject
CPI does not Granger Cause MSPI	14.759	0.002	Reject

AW denotes average wage of urban employees, CPI denotes consumer price index, MSPI_t_ denotes medical service price index, TMSE denotes total medical service expenditure. Rejection of the hypothesis at 5% level

## Discussion

To assess the ongoing medical service prices reform in China, it is necessary to understand historical patterns of medical service price levels from macro-perspective. Our study shows that the health expenditure in China has been increasing along with economic development. From the composition of THE, the absolute number and proportion of TMSE have significantly increased, while the absolute number of TPE has slightly increased but the proportion has significantly decreased. The trends in outpatient and inpatient service volumes are consistent with economic development and health expenditure. Adjusting to the price level in 2000, the price levels in China has been increasing, and the inflation rate of the medical industry is lower than CPI of China. The price levels of medical service has significantly increased, and the inflation rate is higher than the overall price levels in China. However, the price levels of pharmaceuticals is constantly decreasing. Our study further examines the association between TMSE and economic development, price levels, and healthcare utilization. The results suggest that the growth of TMSE after adjusting GDP deflator is driven by prices and health utilization. There is no consensus on whether the rise in health expenses in China is driven by utilization or prices [21], our research from a macroeconomic perspective complements the evidence that the rise in TMSE in China is associated with the increase in the price levels and inpatient utilization of medical service. Previous studies have confirmed the impact of quantity on medical expenditure [3]. The focus of this study is to analyze the role of medical service prices in this context.

Early research on China’s medical pricing management mechanism concluded that the pricing of medical service was significantly below the labor cost of medical staff, while hospitals relied on pharmaceuticals as the primary source of income, leading to a heavy burden on patients [29]. However, this situation was greatly alleviated after the health reform in 2009. Our research findings indicate that the share of drug expenditure has been declining annually while the share of TMSE has been increasing annually. And the gap between the two proportions has been widening year by year. The results align with the policy target of reducing public hospital funding reliance on pharmaceuticals and increasing revenue from medical service. These findings are consistent with the study, which found a substitution effect between pharmaceuticals and medical service [30–31]. The transition of pharmaceuticals from a revenue source to a cost has reduced incentives for hospitals to increase the share of pharmaceuticals.

In China’s health market, patients can freely seek medical treatment, and the government controls medical expenditure growth through prices regulation. Excessive reliance on drug revenue was the fundamental reason for the rapid increase in medical expenses and the launch of China’s 2009 healthcare reform. Therefore, it can be seen that China’s prices regulation policy of reducing generic drug prices from 1997 to 2007, canceling drug markup policies from 2009 to 2017, and the national centralized procurement and price negotiation policies from 2017 to the present have effectively controlled the price levels of drugs during these 21 years.

Compared to pharmaceutical prices, the price levels of medical service has increased nearly twice over 21 years. The strict regulation of medical service pricing in the previous century resulted in distorted medical pricing schemes. Following the introduction of the central government’s inaugural unified medical service catalog in 2001, regions across the country endeavored to synchronize their respective items with the national items. Certain provinces may have retained higher-priced items within the decomposed components and initiated price adjustment measures in this process. Consequently, the price levels of medical service has been experiencing crawling inflation since 2000. Specifically, the decentralization of medical service prices management to the provincial level has led to variations in the timing of alignment among different provinces. The medical service price levels experienced a rapid increase from 2000 to 2007. With the completion of the integration of provincial medical service pricing items, the annual growth rate of medical service price levels has gradually declined from 2007 to 2015. In 2015, China launched a comprehensive reform of public hospitals, which will eliminate medicine prices addition and compensate for the losses by increasing medical service prices. At the same time, the development of the national economy has also provided favorable conditions for reform, with CPI maintaining a relatively low level, bringing a window period for medical service prices adjustment. From 2015 to 2018, the medical service price levels rose rapidly. Since 2018, the reform of drug and consumable markup has been completed, and the prices of medical service has lost this adjustment space, resulting in a slowdown in the rate of prices increases for medical service.

Studying the impact of health expenditure on macroeconomics has policy and academic implications, and further examination is needed to examine the relationship between changes in medical service prices and macroeconomy. This study utilizes the Johansen cointegration test to demonstrate the existence of at least two cointegration relationships between medical service price levels and these macroeconomic factors, indicating a long-run equilibrium relationship between medical service price levels and TMSE, average wage and CPI. After fulfilling the prerequisite of cointegration, Granger causality analysis is used to test the causal relationship between medical service price levels and macroeconomic factors. Changes in the price levels of medical service have an impact on changes in TMSE but have no impact on average wage and CPI. The imperfect mechanism of value transmission between medical service prices and the doctor salary system in China may help explain this finding. Furthermore, the findings indicate the impact of TMSE, AW, and CPI on changes in medical service price levels. Previous research has been unable to ascertain the determinants of medical service pricing [32]. This is primarily due to the decentralized authority for pricing across each province, where prices adjustments may be influenced by the considerations of provincial prices regulators [21]. Our study provides evidence from a macro perspective on the influencing factors of changes in medical service price levels.

The relationship between changes in medical service price levels and average wage, CPI, and TMSE also provides a reference basis for the feasibility of pilot policies for medical service prices reform. The adjustment of medical service pricing has long been criticized for its prolonged and irregular cycles and the absence of a rational triggering mechanism in China [33]. Our research findings provide evidence that the incorporation of lagged macroeconomic indicators offers a systematic approach to guide the adjustment of medical service prices.

There are several limitations to this study. First, this study lacked pure data on medical service expenditure, as medical service price items include medical consumables costs, making it challenging to separate medical consumables expenditure from overall medical service expenditure. While medical service constitute a substantial portion of expenses, our grasp of historical trends in this domain still needs to be improved. Second, the price index we used to reflect price levels is based on a reference basket of goods and service. However, we lacked information regarding the weights assigned to representative items within the basket, and this approach has faced criticism for its limited representation. Third, the pricing and reimbursement systems are independent in China, but health insurance plays a crucial role as the primary payer for medical service in China, changes in the price levels of medical service will have a significant impact on the medical insurance fund. However, China’s social health insurance schemes have undergone rapid reform, including the urban employee basic medical insurance scheme (UEBMI; launched in 1998), the rural new cooperative medical scheme (NCMS; launched in 2003), the urban resident basic medical insurance scheme (URBMI; launched in 2007), and the urban-rural resident basic medical insurance scheme (URRBMI; launched in 2017), which merged the original urban resident basic medical insurance and rural new cooperative medical scheme. Historical data on health insurance funds are difficult to obtain. Therefore, we could not further investigate the relationship between medical service prices and medical insurance. These limitations should be considered when interpreting our findings.

## Conclusions

This study assessed the time trend of medical service price levels and the relationship with relevant macroeconomic factors since the beginning of this century. The growth of medical service expenditure in China was driven by the increase in inpatient use and price level of medical service and medicine. From a macro perspective, the change in price levels of medical service had an impact on medical service expenditure, but had no impact on average wage of employees and CPI. The value transmission mechanism of medical service price policies needs further optimization.

## Data Availability

The datasets used or analysed during the current study are available from the corresponding author on reasonable request.

## Abbreviations

THE: Total health expenditure
TMSE: Total medical service expenditure
TPE: Total pharmaceuticals expenditure
GDP: Gross domestic product
CPI: Consumer price index
HPI: Health care price index
MPI: Medicine price index
MSPI: Medical service price index
